# Microwave Properties of Coplanar Waveguide-Based PEDOT:PSS Conducting Polymer Line in Ethanol Gas Atmosphere

**DOI:** 10.3390/ma13071759

**Published:** 2020-04-09

**Authors:** Hee-Jo Lee, Nathan Jeong, Hyang Hee Choi

**Affiliations:** 1 Department of Physics Education, Daegu University, Gyeongsan 38453, Korea; 2 School of Electrical and Computer Engineering, University of Alabama, Tuscaloosa, AL 35487, USA; shjeong@ua.edu; 3 Department of Chemical and Biomolecular Engineering, Yonsei University, Seoul 03722, Korea; netchoi@yonsei.ac.kr

**Keywords:** coplanar waveguide, poly(3,4-ethylenedioxythiophene) polystyrene sulfonate (PEDOT:PSS), conducting polymer, gas sensor, ethanol gas

## Abstract

This study aims to investigate the microwave properties of coplanar waveguide (CPW)-based poly(3,4-ethylenedioxythiophene) polystyrene sulfonate (PEDOT:PSS) conducting polymer line in an ethanol gas atmosphere, with the frequency range of 0.5–2 GHz. For an ethanol-exposed PEDOT:PSS line (test sample), the transmission coefficient (*S*_21_) decreased immediately; moreover, the microwave effective conductivity (σ*_m/w_*) decreased simultaneously, compared with the ethanol-free PEDOT:PSS line (reference sample). The immediate variations in Δ*S*_21_ ( = *S*_21,ethanol_ − *S*_21,free_) and Δσ*_m/w_* ( = σ*_m/w_*_,ethanol_ − σ*_m/w_*_,free_) were approximately 10.2 dB and 2.7 × 10^4^ S/m, respectively. Furthermore, in the analysis of the circuit model of the PEDOT:PSS line, the characteristic impedance and distributed elements, i.e., resistance (*R*) and inductance (*L*) per length, of the test sample increased, compared with the reference sample. However, upon stopping the exposure to ethanol gas, the microwave properties of the test sample instantaneously recovered to those of the reference sample. According to these critical observations, we could confirm that the coplanar waveguide with a PEDOT:PSS line shows a significant difference in the diverse microwave properties, through rapid response to the ethanol gas at room temperature.

## 1. Introduction

Polymers have typically been used as insulating materials, but with the discovery of conducting polymers (CPs), they have been used as conductive materials as well. Since then, CP materials have received tremendous attention from researchers to explore their fundamental properties and applications because of the similarity of their electrical properties to those of metals and semiconductors [1]. In particular, the presence of conjugated π-electrons in CPs confers unique electrical and optical properties, including low ionization potential, high electron affinity, and low energy optical transition [2,3]. First of all, these properties were used to enhance the performance of sensors relaying on various transducers [4,5,6,7], e.g., potentiometric, amperometric, piezoelectric, calorimetric, thermal, and optical mode. The new CP sensor applications [8,9,10], including electrochemical and biosensors, exhibited excellent characteristics in terms of sensitivity.

Owing to the CPs processability and metallicity, some researchers conducted studies on microwave conductivity-based technology and its applications, such as electromagnetic interference shielding [11], electrostatic charge dissipation or antistatic [12], microwave absorption [13], and radar cross-section reduction [14]. However, the existing CPs, used for microwave applications, were limited by their low electrical conductivity.

In recent years, the usage of gas [15,16] and humidity [17] sensors has become more feasible in microwave applications because of the techniques [18,19] that have been developed to enhance the electrical conductivity of CPs. However, the basic mechanism of interaction between a specific gas and an active material has not been adequately researched; it is necessary to address this gap to improve the performance of microwave sensors. In addition, there is still room to improve sensitivity in terms of the microwave properties, which include S-parameter, characteristic impedance, microwave effective conductivity, and others.

In this work, ethanol gas will be used as an interacting gas to the active material, PEDOT:PSS line, because this gas is closely related to food, e.g., fruits ripening [20] and plant growth [21], as well as public health, e.g., sterilization [22]. Thus, we investigate the microwave properties of the coplanar waveguide (CPW) based on the PEDOT:PSS CP line in an ethanol atmosphere. Furthermore, we undertake an in-depth analysis of the interaction mechanism between the ethanol gas and the PEDOT:PSS line in the frequency region of 0.5–2 GHz. Finally, we demonstrate that a coplanar waveguide with a PEDOT:PSS line can produce significant differences in the diverse microwave properties of an ethanol gas atmosphere at room temperature.

## 2. Experiment 

### 2.1. Design and Simulation

**Figure 1:**
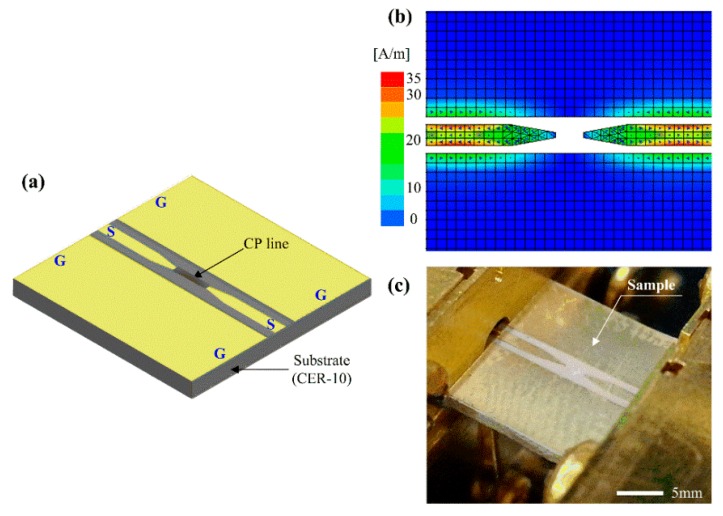
Design, simulation, and fabrication of a bare coplanar waveguide (CPW) device; (**a**) Schematic of the CPW device with a PEDOT:PSS line. The overall area of the CPW device is 10 mm × 10 mm; (**b**) Average surface current distribution at 2 GHz; (**c**) Sample image of the bare CPW device.

To examine the microwave properties of a PEDOT:PSS film (line) with and without ethanol gaseous exposure, a CPW device with a ground (G)-signal (S)-ground (G) electrode was employed, as shown in Figure 1a.

Here, the CPW device was used as a type of transmission line that can generate a quasi-transverse electromagnetic (TEM) mode between the ground and signal electrodes. Firstly, the CPW without a PEDOT:PSS line (a bare CPW) was designed with a 50 Ω-impedance for transporting maximum power from the microwave source. Subsequently, to verify the power transport on the bare CPW, this was simulated with a 2.5D full-wave electromagnetic solver, based on the method of moments (MoM).

Figure 1b shows the average surface current distribution as the degree of microwave power transport, i.e., 50 Ω-impedance matching, in the bare CPW. As can be seen from the figure, the current becomes concentrated in the region between the ground and signal electrodes when the quasi-TEM mode is generated through the structure. Figure 1c depicts the sample image of the bare CPW through a commercially printed circuit board technique [23].

### 2.2. Response Mechanism between PEDOT:PSS and Ethanol Gas Molecule

Gas molecules can be controlled by acid/base reactions. This reaction mechanism accounts for CPs becoming acidic/basic analytes as their conductivity changes (doping/dedoping process). There are also proposed sensing mechanisms for conductive polymer systems, including redox reactions between polymers and analytes, charge transfer between polymers and analytes, and polymer swelling. The resistance of PEDOT:PSS increases upon ethanol exposure and decreases to its initial baseline in pure air. This resistance-changing behavior may be explained by ethanol sensing reactions.

O_2_ (gas) → O_2_ (phys) → O^−^_2_ (chem)

C_2_H_5_OH → CH_3_CHO + H_2_

2CH_3_CHO (ad) + 5 O^−^_2_ → 4CO_2_ + 4 H_2_O + 5e^−^

It is known that O^−^_2_ is the predominant chemisorbed oxygen species on some active materials at room temperature. The adsorbed oxygen is mainly in the form of O^−^_2_ below the temperature of 100 °C. Therefore, when exposed to ethanol, C_2_H_5_OH molecules subsequently dissociate into CH_3_CHO and interact with the adsorbed oxygen to form CO_2_ and H_2_O. When ethanol molecules are adsorbed on the PEDOT:PSS surface by physisorption, the holes of the conductive PEDOT:PSS interact with the electron-donating ethanol analyte. In the case of PEDOT:PSS sensing materials, they act as conductive pathways that favor the hopping of electrons. The increase of PEDOT:PSS distance pathways occurs simultaneously, leading to a significant increase of the PEDOT:PSS upon ethanol exposure and, therefore, enhanced ethanol response.

### 2.3. Sample Preparation and Measurement System

**Figure 2:**
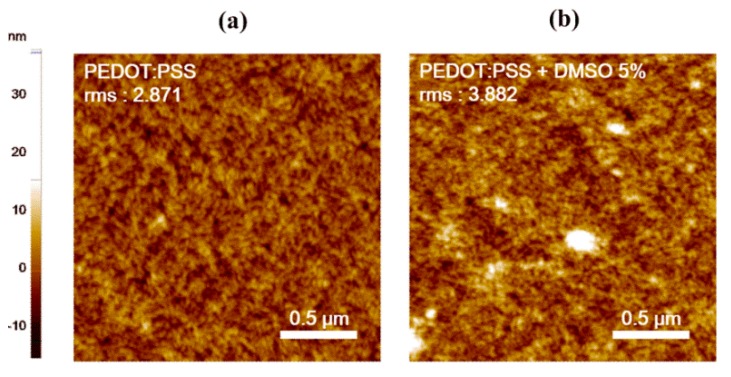
AFM images of PEDOT:PSS films: (**a**) pristine PEDOT:PSS and (**b**) PEDOT:PSS with DMSO.

In the present experiment, the CPW with a PEDOT:PSS line was prepared as follows. First of all, for the PEDOT:PSS line, a PEDOT:PSS (Clevios PH 1000) and dimethyl sulfoxide (DMSO) (99%) solution were purchased from Heraeus, (Hanau, Germany) and Sigma-Aldrich (St. Louis, MO, USA), respectively. Here, all reagents were used without any treatment. Subsequently, the PEDOT:PSS solution was doped with 5 wt.% DMSO solvent. The doped PEDOT:PSS can operate as a highly sensitive material to ethanol gas because electron transfer easily occurs through π-orbital overlap. In the case of PEDOT:PSS, electron transfer occurs through π-orbital overlap, when single and double bonds of a typical carbon atom of a conductive polymer cross when treated with a solvent. Therefore, the overlap of π-orbitals becomes stronger, so the electron movement is much faster. The topographical difference in PEDOT:PSS, treated with and without DMSO, was observed using an atomic force microscope (AFM) (Park systems, Suwon, South Korea), as shown in Figure 2a,b.

**Figure 3:**
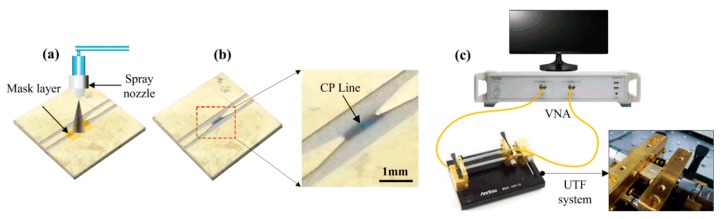
Sample preparation and experimental system for sample measurement; (**a**) Spray method for PEDOT:PSS line; (**b**) Pattern of the PEDOT:PSS line between the signal (S) electrodes; (**c**) Experimental system for sample measurement.

The surface roughness of the PEDOT:PSS films, treated with and without DMSO, was approximately 3.882 and 2.871 root-mean-square (rms), respectively. Next, the bare CPW was treated with oxygen plasma for 3 min to enhance the hydrophilicity of the surface. After that, a PEDOT:PSS solution doped with DMSO was sprayed, as shown in Figure 3a.

Here, the dimensions of the patterned PEDOT:PSS line were approximately 1.2 mm long, 0.14 mm wide, and 8.0 μm thick. Subsequently, the CPW with a PEDOT:PSS line was annealed on a hot plate at 100 °C for 5 min at ambient conditions. Finally, Figure 3b shows the sample image of the CPW with a PEDOT:PSS line.

Figure 3c depicts the experimental setup, consisting of a universal test fixture (UTF) (3680 Series, Anritsu, Atsugi, Japan) and a two-port vector network analyzer (VNA) (MS46322A, Anritsu, Atsugi, Japan) for sample measurement. The frequency range was set from 0.5 to 2 GHz, and the number of points and input power were set to be 101 and −20 dB, respectively. After that, the measurement system was calibrated by a short-open-load-through (SOLT) method with a calibration kit (36804B-15M, Anritsu, Atsugi, Japan). Moreover, the CPW with a PEDOT:PSS line in the UTF system was tested in an ethanol atmosphere of 100 ppm concentration at a temperature of 25 °C and relative humidity of 65%. Here, the ethanol gas was regularly exposed with a volumetric flow rate of 1000 cc/min through a flow meter, and, simultaneously, nitrogen was used as a carrier gas.

## 3. Results and Discussion

### 3.1. Transmission Coefficient and Microwave Effective Conductivity

**Figure 4:**
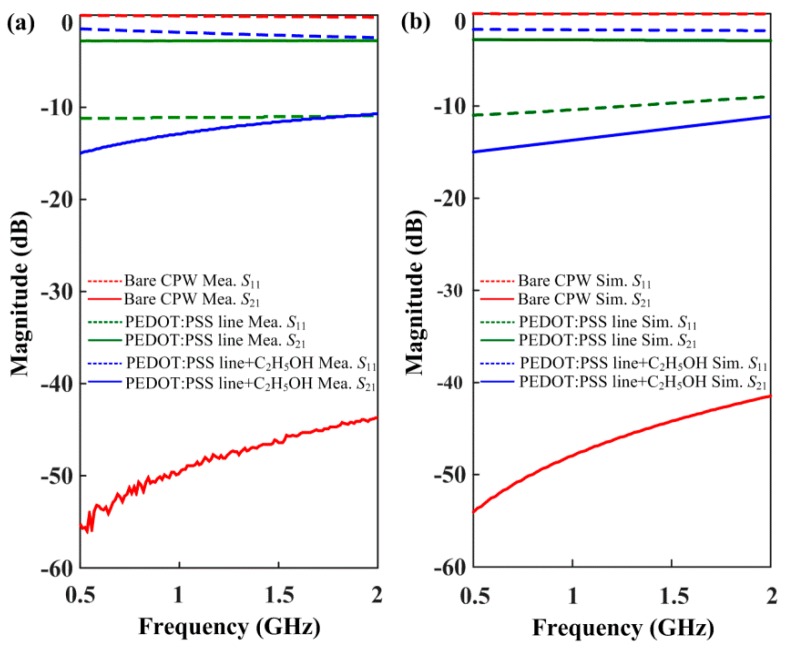
Magnitude of S-parameters in the three sample configurations; (**a**) Measured; (**b**) Simulated samples.

Figure 4a,b depicts the magnitude of the *S*-parameters of the simulated and measured samples at three sample configurations—a bare CPW, a CPW with a PEDOT:PSS line (ethanol-free PEDOT:PSS line), and a CPW with a PEDOT:PSS line exposed to ethanol gas (ethanol-exposed PEDOT:PSS line).

The magnitude of the *S*_11_- and *S*_21_-parameters can be expressed as follows, Equation (1):

(1) $$S_{11}=20\log\left(\frac{V_{1,out}}{V_{1,in}}\right),S_{21}=20\log\left(\frac{V_{2,out}}{V_{1,in}}\right)$$

where *S*_11_ and *S*_21_ are the reflection and transmission coefficients of voltage waves, respectively. Here, *V*_1,in_ and *V*_1,out_ indicate the input- and output-voltage waves, respectively, at the first-port, and *V*_2,ou_ indicates the output-voltage wave at the second port of the microwave VNA source. As shown in Figure 4a, the bare CPW exhibited the typical property of a microwave capacitor; this can be seen by the increasing *S*_21_-level at higher frequencies, i.e., it shows the *S*_21_-level from −56 dB (at 0.5 GHz) to −45 dB (at 2 GHz). For the ethanol-free PEDOT:PSS line, the *S*_21_-level indicated −2.8 dB uniformly, which is much higher (approximately 130 times) than the level in the bare CPW in the observed frequency range. Therefore, it is noticeable that the PEDOT:PSS film having a conductive property can be regarded as the transmission line (TL) circuit between the signal electrodes. On the other hand, the ethanol-exposed PEDOT:PSS line showed the *S*_21_-level from −15 dB (at 0.5 GHz) to −11 dB (at 2 GHz). The Δ*S*_21_ responding difference between the ethanol-free and the ethanol-exposed PEDOT:PSS line corresponds to 12.2 dB (at 0.5 GHz) and 8.2 dB (at 2 GHz), approximately. This response can be regarded as a very significant variation when using ethanol gas of 100 ppm concentration.

Meanwhile, to evaluate the microwave effective conductivity (σ*_m/w_*) of the PEDOT:PSS line itself, the two kinds of samples—an ethanol-free PEDOT:PSS line (reference sample) and an ethanol-exposed PEDOT:PSS line (test sample)—were measured with and without exposure to ethanol gas. Here, the microwave effective conductivity (σ*_m/w_*) is defined as $1/R_{s}\delta$, where $R_{s}$ and δ are the sheet resistance and penetration depth, respectively. The σ*_m/w_* of each sample was estimated by the fitting method, based on the results measured with electromagnetic simulation under the conductivity variable. Figure 4b shows the fitted results obtained from the electromagnetic simulation. The σ*_m/w, free_* of the ethanol-free PEDOT:PSS line was approximately 3.0 × 10^4^ S/m, which corresponds to the −2.8 dB result on the *S*_21_-level. Further, the fitted σ*_m/w,ethanol_* of the ethanol-exposed PEDOT:PSS line was 2.5 × 10^3^ − 3.0 × 10^3^ S/m, which corresponds to the *S*_21_-level range from −15 dB (at 0.5 GHz) to −11 dB (at 2 GHz). Here, the conductive range results from the discrepancy between the measured and the fitted results. However, as shown in Figure 4, these results were in good agreement with the measured results.

Notably, when the ethanol gas was removed entirely, the *S*_21_-level recovered immediately to the initial *S*_21_-level of −2.8 dB, i.e., to the ethanol-free PEDOT:PSS line. According to these results, we found that the *S*_21_-parameter of the PEDOT:PSS line exhibits the real-time response with and without exposure to ethanol gas. Notably, the exposure of ethanol gas can result from the degradation of the microwave effective conductivity of the PEDOT:PSS line, i.e., increasing the surface resistance. This is because the carriers, including *π*-electrons of the PEDOT:PSS line, have low mobility [24] when the ethanol gas, the hydroxyl radicals in the ethanol gas molecules, and the ethyl group linked to the hydroxyl (–OH) group [8], selectively absorb the PEDOT:PSS line. This effect is instantaneously caused by the high surface resistance.

### 3.2. Characteristic Impedance and Distributed Elements

**Figure 5:**
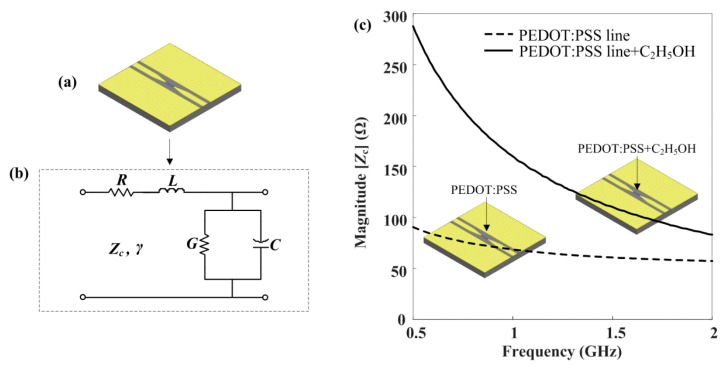
Impedance and distributed elements of the PEDOT:PSS line; (**a**) Schematic of CPW with a PEDOT:PSS line, (**b**) Transmission line (TL) circuit model of the CPW with a PEDOT:PSS line, (**c**) Magnitude of characteristic impedance.

Figure 5a depicts the CPW with a PEDOT:PSS line. As shown in Figure 5b, the PEDOT:PSS line can be modeled as a TL circuit, including resistance (*R*), inductance (*L*), conductance (*G*), and capacitance (*C*) per unit length (mm).

Furthermore, the characteristic impedance (*Z*_c_) and propagation constant (γ) can be expressed as the distributed elements, Equation (2):

(2) $$Z_{c}=\sqrt{\left(R+j\omega L)/(G+j\omega C\right)},\;\gamma =\sqrt{\left(R+j\omega L)(G+j\omega C\right)},$$

where *ω* is the angular frequency. In Figure 5c, the magnitude of *Z*_c_ ($\left|Z_{c}\right|$) of the ethanol-exposed PEDOT:PSS line was higher than that of the ethanol-free PEDOT:PSS line in the observed frequency region.

**Figure 6:**
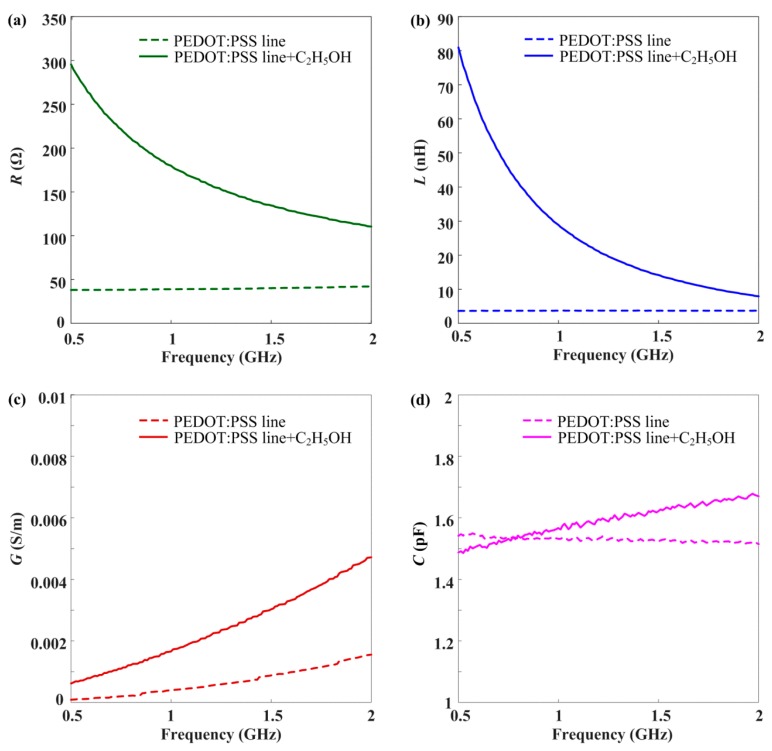
Difference of distributed elements of the PEDOT:PSS line with and without ethanol gas; (**a**) *R*; (**b**) *L*; (**c**) *G*; (**d**) *C* component.

However, the $\left|Z_{c}\right|$ of the ethanol-exposed sample dramatically decreased with increasing frequency. In addition, these samples gradually converged near 2 GHz. Figure 6a–d shows the difference of the distributed elements of the PEDOT:PSS line with and without ethanol gas in the observed frequency region.

These components can be obtained from the relationship between the ABCD matrix and the *S*-parameter, as follows [25]:

(3) $${\left[\begin{array}{cc} A & B\\ C & D \end{array}\right]}_{P.S.}=\left[\begin{array}{cc} \frac{1-S_{11}^{2}+S_{21}^{2}}{2S_{21}} & \frac{{\left(1+S_{11}^{}\right)}^{2}-S_{21}^{2}}{2S_{21}Z_{0}}Z_{0}\\ \frac{{\left(1-S_{11}^{}\right)}^{2}-S_{21}^{2}}{2S_{21}Z_{0}} & \frac{1-S_{11}^{2}+S_{21}^{2}}{2S_{21}} \end{array}\right]$$

(4) $${\left[\begin{array}{cc} A & B\\ C & D \end{array}\right]}_{P.S.}=\left[\begin{array}{cc} \cosh(\gamma l) & Z_{c}\sinh(\gamma l)\\ \frac{\sinh(\gamma l)}{Z_{c}} & \cosh(\gamma l) \end{array}\right]$$

Using Equations (2)–(4), the electric elements of the TL circuit model can finally be derived as follows:

(5) [RLGC]=[Re[γZc]Im[γZc]/ωRe[γ/Zc]Im[γ/Zc]/ω]

From Equation (5), the distributed components to the ethanol-free and ethanol-exposed PEDOT:PSS line were obtained. The series *R* and *L* components of the ethanol-exposed PEDOT:PSS line were significantly reduced, as shown in Figure 6a,b. However, the corresponding components of the ethanol-free PEDOT:PSS line were uniform in the observed frequency region. Moreover, the shunt *G* and *C* components of the ethanol-exposed PEDOT:PSS line increased gradually compared with those of the PEDOT:PSS line, as shown in Figure 6c,d. According to these results, we found that the *R* and *L* components exhibit a considerable difference as sensing components with and without exposure to ethanol gas.

## 4. Conclusions

In this study, we have observed a degradation of the *S*_21_-level and microwave effective conductivity of the PEDOT:PSS line, with and without exposure to ethanol gas, in the observed frequency region of 0.5–2 GHz. In particular, upon exposure to ethanol gas, the *S*_21_-level on the PEDOT:PSS line was lowered by approximately 25% in comparison to that of the ethanol-free PEDOT:PSS line. Moreover, this decreased the microwave effective conductivity by approximately 10 times, matching with that of the ethanol-free PEDOT:PSS line. Using the TL circuit model of the CPW with a PEDOT:PSS line, we have demonstrated a remarkable difference in the microwave properties, including the distributed elements and characteristic impedance. In particular, the *R* and *L* components of the PEDOT:PSS line, with and without exposure to ethanol gas, exhibited a considerable difference of less than 2 GHz. In the analysis of the microwave TL circuit, we demonstrated that a low carrier mobility also exhibits the increase of *R* and *L* components because it binds ethanol gas molecules onto the surface of the PEDOT:PSS line. Consequently, we could confirm that ethanol gas not only influences the degradation of the microwave properties of the PEDOT:PSS line itself, but also causes this to occur very rapidly. These properties can provide a new alternative for developing highly sensitive and robust PEDOT:PSS-based microwave gas sensor platforms in the future.
